# Characterization of the Human Plasma Biofilm Model (hpBIOM) to Identify Potential Therapeutic Targets for Wound Management of Chronic Infections

**DOI:** 10.3390/microorganisms12020269

**Published:** 2024-01-26

**Authors:** Michael Dietrich, Manuela Besser, Ewa Klara Stuermer

**Affiliations:** 1 Institute of Virology and Microbiology, Centre for Biomedical Education and Research (ZBAF), Faculty of Health, Witten/Herdecke University, 58455 Witten, Germany; 2 Department of Vascular Medicine, University Heart and Vascular Center, University Medical Center Hamburg-Eppendorf (UKE), 20246 Hamburg, Germany

**Keywords:** wound biofilm, MRSA, *S. aureus*, *P. aeruginosa*, wound infection, host immune system

## Abstract

The treatment of chronic wounds still represents a major challenge in wound management. Recent estimates suggest that 60–80% of chronic wounds are colonized by pathogenic microorganisms, which are strongly considered to have a major inhibiting influence on the healing process. By means of an innovative biofilm model based on human plasma, the time-dependent behavior of various bacterial strains under wound-milieu-like conditions were investigated, and the growth habits of different cocci species were compared. Undescribed fusion events between colonies of MRSA as well as of *Staphylococcus epidermidis* were detected, which were associated with the remodeling and reorganization of the glycocalyx of the wound tissue. After reaching a maximum colony size, the spreading of individual bacteria was observed. Interestingly, the combination of different cocci species with *Pseudomonas aeruginosa* in the human plasma biofilm revealed partial synergistic effects in these multispecies organizations. RT-qPCR analyses gave a first impression of the relevant proteins involved in the formation and maturation of biofilms, especially the role of fibrinogen-binding proteins. Knowledge of the maturation and growth behavior of persistent biofilms investigated in a translational human biofilm model reflects a starting point for the development of novel tools for the treatment of chronic wounds.

## 1. Introduction

The development of chronic wounds is often accompanied by infection with pathogenic microorganisms forming a biofilm [1,2,3]. Biofilms are composed of microorganisms surrounded by a complex matrix of carbohydrate polymers (glycocalyx), proteins and eDNA, known as the extracellular polymeric substance (EPS) [4,5,6]. The EPS aggravates the biofilm treatment by means of antimicrobial solutions. Additionally, the effect of antibiotics is inhibited due to the reduced metabolism of the pathogens in biofilms [7,8,9,10]. Bacteria can communicate with each other or with their host environment, known as quorum sensing (QS), to exchange information, for instance, about cell density or nutrient supply, allowing the microorganisms to act like multicellular organisms [11,12,13]. In addition, biofilms bear the intriguing capacity to alter the human immune response [14,15,16].

A wide variety of bacterial virulence factors are involved in all these processes. Of particular importance for the initial formation and maturation of biofilms is the binding to both human cell components as well as between the bacteria among themselves. Familiar representatives for binding proteins are the so-called clumping factors, like clumping factor A (ClfA), which are upregulated during biofilm maturation [17,18]. They ensure the agglutination of *S. aureus* with fibrinogen in solution and the binding to immobilized fibrinogen [17,19,20]. Other examples are the accumulation-associated protein (Aap) of *S. epidermidis*, which can become an intercellular adhesin [21,22], and the extracellular matrix-binding protein (Embp) that has fibronectin-binding activity and is expressed predominantly during the growth phase [23,24]. The increased expression of Embp as well as the release of eDNA during biofilm formation is caused by the virulence factor SarA as part of a QS process [25,26,27].

Equally important as the binding is the protection against the human immune response. Neutrophilic leukocytes and macrophages are the main immune responses against bacteria [28,29,30]. A part of the immune response involves the release of matrix metalloproteases (MMPs), and neutrophils produce large amounts of reactive oxygen intermediates [31,32]. They can also secrete various proinflammatory cytokines, such as interleukin-1 beta (IL1-β) [29,33]. In response, bacteria secrete various neutrophil toxins [34,35,36]. The expression of ClfA significantly protects *S. aureus* from macrophage phagocytosis. This is also true for Aap and EmbP, which both protect *S. epidermidis* biofilms from phagocytic uptake by macrophages [14]. Another defense mechanism is the competition for iron. Bacteria use different molecules to acquire iron from their hosts [37]. Proteins like the cell wall protein iron-regulated surface determinant A (IsdA) of *S. aureus* is in turn expressed under iron-limited conditions [38,39,40]. Its inactivation induces a decrease in skin colonization by *S. aureus* [38]. Like ClfA or Embp, IsdA also binds to human fibrinogen and fibronectin, thereby providing a defense against the skin’s innate immune response [38,41].

Our working group established a novel human biofilm model recently, based on coagulated human plasma inoculated with bacteria. The human plasma biofilm model (hpBIOM) mimics a wound environment suffering from a biofilm infection [42]. This model has already provided new insights into the mode of action of antiseptics on biofilms. The test of clinical standard applications displayed significantly reduced efficacy in the eradication of the bacteria of the hpBIOM [43]. The validation of existing therapeutics is a possible application of this model. A major challenge in the current guideline development is the transfer of data based on in vitro biofilm models developed on solid surfaces (glass slides or cell culture wells) to the clinical wound situation. In contrast, the hpBIOM and comparable models present the adhesion possibilities and the milieu of acute and chronic wounds, which make it more suitable for translational wound research [44].

The aim of this work was to characterize the human plasma biofilm model in more detail regarding the bacterial growth and communication behavior and the interaction with the individual hosts’ immune response.

The following questions were raised: How do bacteria grow in this model? Are their differences in the expression of basic genes implied in the context of adhesion, interaction or quorum-sensing processes, respectively? Does the donor have an influence on the behavior, and can new treatment recommendations be derived from these findings in the context of personalized medicine?

## 2. Materials and Methods

### 2.1. Bacteria Strains

*Staphylococcus epidermidis* (DSM-20044), *Enterococcus faecium* (DMS-2146) and *Pseudomonas aeruginosa* (DSM-939) were obtained from ATCC. Methicillin-resistant *Staphylococcus aureus* (MRSA) was a clinical isolate, kindly provided by Beniam Ghebremedhin (Helios University Hospital, Wuppertal, Germany). All strains were cultured on casein/soy peptone agar plates (CSA; pH 7.2) with 15 g/L casein peptone, 5 g/L soy peptone, 5 g/L sodium chloride and 15 g/L agar (AppliChem, Darmstadt, Germany) under aerobic conditions at 37 °C.

### 2.2. Preparation of the Human Plasma Biofilm Model (hpBIOM)

Plasma and buffy coats from anonymous donors were obtained from DRK-Blutspendedienst West (Hagen, Germany). Remaining erythrocytes in the buffy coat were removed via centrifugation for 30 min at 1610× *g* at room temperature. Plasma and buffy coats were combined, carefully mixed in a sterile glass bottle and shaken continuously at 22 °C. Each model consisted of 1.5 mL of this solution inoculated with 1 × 10^6^ colony-forming units (cfu) pathogenic bacteria in single species biofilm and with 2 × 10^5^ cfu each in mixed species biofilms (*P. aeruginosa* + MRSA or *P. aeruginosa* + *E. faecium*). The donated blood plasma contained sodium citrate as the standard to inhibit blood coagulation. Therefore, the antagonist calcium chloride (CaCl_2_; 6.1 µM) was added to induce the polymerization of the plasma. The mixture was immediately transferred to 12-well culture plates (Sarstedt AG & Co., Nürnbrecht, Germany). The plates were incubated for 1–72 h on a rotary shaker at 50 rpm and 37 °C. Polymerization formed a stable biofilm clot with integrated pathogens, which was applied for the analysis.

### 2.3. Histology and Immunohistochemistry

After incubation for 1 h, 12 h and 24 h, as well as 48 h and 72 h for *S. epidermidis* and MRSA, the stable biofilm clots were gently taken completely out of the culture plates and were fixed overnight in 4% paraformaldehyde. After fixation, the hpBIOMs were embedded in paraffin. At intervals of 0.25 mm 10 µm thick microtome sections were prepared and stored on glass slides at RT. For histomorphological examinations, the samples were deparaffinized and rehydrated via ethanol/xylene washing steps and subjected to hematoxylin and eosin staining. H/E staining was chosen to achieve a comprehensive overview of the fibrin structures, immune cells, and the bacteria. This method was applied because, by means of gram-staining, no further information regarding the histomorphology could be gained. The H/E staining enabled the examination of bacterial colonies.

To determine the colony size, a minimum of 5 non-consecutive sections were selected from at least two hpBIOMs per donor per time point, and all colonies in ten image sections were analyzed via microscopy. The length and width of the colonies were measured via Leica Application Suite X (LAS-X) (Leica Mikrosysteme Vertrieb GmbH, Wetzlar, Germany), and the diameter was calculated based on the average.

For the detection of the glycocalyx, the sections were incubated with 50 µg/mL Concanavalin A (Sigma-Aldrich, St. Louis, MO, USA), 20 µM SYTO^®^Red (Molecular Probes/Carl Roth) and Roti^®^-Mount FluorCare DAPI (Carl Roth, Karlsruhe, Germany) in a blocking solution consisting of 1× PBS, 1% BSA, 0.1% Triton X100, 0.1% Tween 20 and 5% goat serum (Pan Biotech, Aidenbach, Germany) in a humidified chamber at room temperature for 30 min.

### 2.4. RNA and Protein Isolation from hpBIOMs

Like in Section 2.3, the samples were gently removed from the culture plates after incubation and immediately stored at −80 °C. For the isolation of RNA and proteins from the samples, 1 mL of a 10% bromelain/PBS solution (Bromelain-POS, RSAPHARM Arzneimittel GmbH, Saarbrücken, Germany) was applied to each clot after a two-hour thawing process on ice. The enzymatic digestion of the hpBIOM was kept for 1 h at RT whilst resuspending several times. The suspensions as well as 2 mL of planktonic bacterial suspensions (OD^600^: 0.1) as 0 h controls were centrifugated at 15,000× *g* for 5 min at 4 °C. The supernatants were discarded, and DNA, RNA and proteins were isolated from the pellets using the AllPrep^®^ Bacterial DNA/RNA/Protein Kit (Qiagen, Hilden, Germany) according to the manufacturer’s instructions. The RNA and protein samples were stored at −20 °C. The RNA concentration was photometrically determined by measuring the absorbance in the UV range at 260 and 280 nm with the Nano-Photometer p330 (Implen, Munich, Germany).

### 2.5. RT-qPCR

The isolated hpBIOM RNA was synthesized into complementary DNA (cDNA) with the First Strand cDNA Synthesis Kit (Invitrogen, Waltham, MA, USA). The RT-qPCR was performed with the primers listed in Table 1 in a CFX96™ Real-Time System (Bio-Rad, Hercules, CA, USA) using the iTaqTM Universal SYBR^®^ Green Supermix (Bio-Rad, Hercules, CA, USA) for detection. Previously, the optimal annealing temperature for the samples was determined by applying a temperature gradient. For the normalization of the results, the expression of the *DNA gyrase subunit β* in *S. aureus* or *S. epidermidis* was used as a reference.

### 2.6. Enzyme-Linked Immunosorbent Assay for Quantitative Detection of Human IL-1β

The isolated proteins from hpBIOMs containing MRSA, *S. epidermidis*, and from control plasma clot samples without bacteria after 1, 24, 48 and 72 h of maturation were analyzed photometrically at 450 nm using the Human IL-1β ELISA Kit (Invitrogen, Waltham, WA, USA) according to the manufacturer’s instructions. The Il-1β concentration was subsequently determined by calibration degrees.

### 2.7. Statistics

Data are expressed as means ± standard deviation (SD). For the biofilm evaluation, triplicates of three different anonymous blood donors were analyzed using the statistics program GraphPad PRISM (Version 8.2.1; GraphPad Software Inc., La Jolla, CA, USA). Statistical analysis contained a one-way ANOVA, followed by Tukey’s post hoc test for the evaluation of multiple comparisons. A *p*-value of *p* ≤ 0.05 was considered statistically significant.

## 3. Results

**Figure 1:**
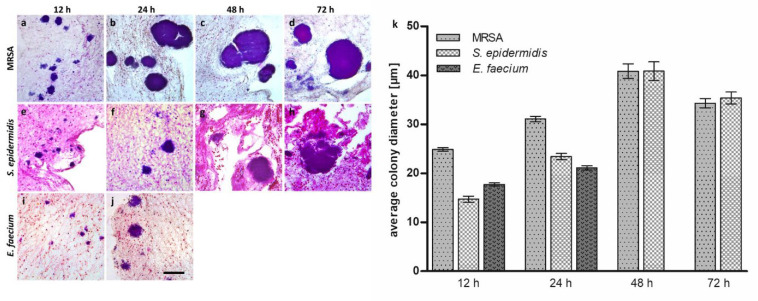
Time-dependent progression of bacterial growth of various cocci species in the hpBIOM. Hematoxylin–eosin-stained hpBIOMs of MRSA (**a**–**d**), *S. epidermidis* (**e**–**h**) and *E. faecium* (**i**,**j**) after 12, 24, 48 and 72 h of maturation (scale bar: 50 µm). (**k**) Average colony diameter of MRSA (dots), *S. epidermidis* (squares) and *E. faecium* (triangles) colonies after 12, 24, 48 and 72 h of incubation. Due to advanced hpBIOM degradation, no further sampling was carried out for *E. faecium* after 24 h. (Values expressed as mean ± SD; statistical analyses revealed no significant differences).

**Figure 2:**
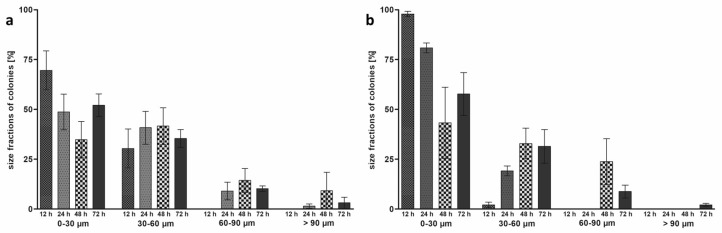
Size distribution of MRSA and *Staphylococcus epidermidis* colonies in the hpBIOM. Fractions [%] of colonies with diameters of 0–30 µm, 30–60 µm, 60–90 µm and >90 µm after 12, 24, 48 and 72 h with MRSA (**a**) and with *S. epidermidis* (**b**). (Values expressed as mean ± SD; statistical analyses revealed no significant differences).

For the morphological examination of the bacteria in the hpBIOM, histological samples were prepared and visualized via hematoxylin and eosin staining. In the light microscopy images of the different coccus species in the hpBIOM, all three species, MRSA, *S. epidermidis* and *E. faecium*, were detected, and the formation of colonies within the first 12 h was observed (Figure 1a,e,i). The time-dependent average colony diameters are plotted in Figure 1k. MRSA showed the largest and *S. epidermidis* the smallest average colony diameter after 12 h. All colonies increased in size within the next 12 h. MRSA developed the largest colonies, while *S. epidermidis* surpassed *E. faecium* in size, although both remained below the average diameter of MRSA after 12 h. No hpBIOM of *E. faecium* could be investigated after 24 h due to degradation processes. MRSA and *S. epidermidis* reached their maximum average colony diameter after a maturation period of 48 h, followed by a slight decrease in size after 72 h. *S. epidermidis* colonies reached their absolute maximum size of >90 µm after 72 h (Figure 2b). An increasing number of small colonies with diameters of 0–30 µm were detected after 48 h and 72 h, which reduced the average diameter of all colonies. For *S. epidermidis*, no colony formation was observed in approx. 25% of all hpBIOMs. The number and the maximum size of the colonies were strongly influenced by the immune cells of the blood donors with a high similarity for MRSA and *S. epidermidis* regarding the growth behavior after the first 48 h (Figure 1). The appearance of small colonies (0–30 µm diameter) after 72 h compared to 48 h was also consistent within the species.

**Figure 3:**
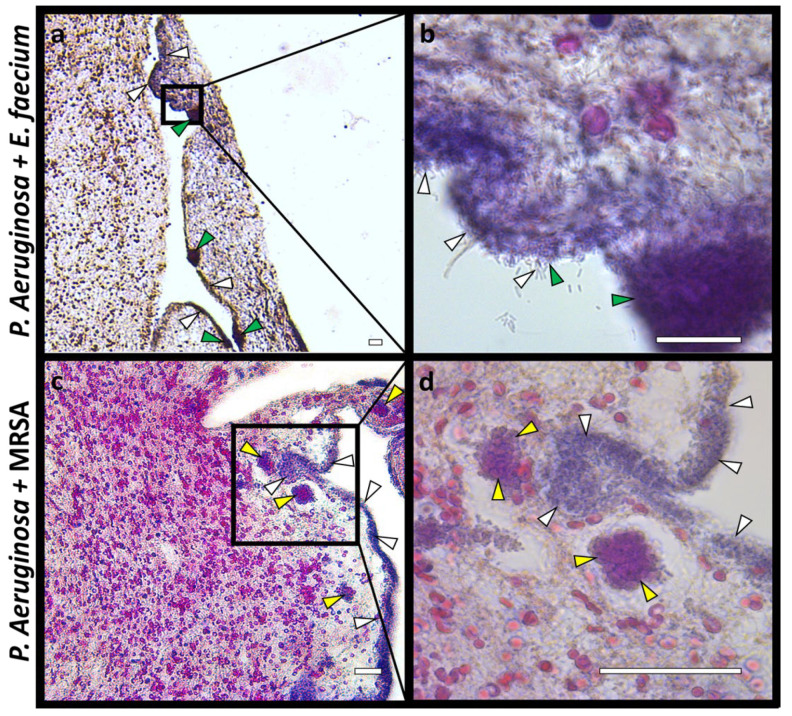
Histological overview and detailed images of multispecies hpBIOMs. Hematoxylin–eosin staining of 24 h matured hpBIOMs with *P. aeruginosa* combined with *E. faecium* (**a**,**b**) and *P. aeruginosa* and MRSA (**c**,**d**) (*P. aeruginosa*, white arrows; *E. faecium*, green arrows; and MRSA, yellow arrows). (Scale bar: 25 µm).

The study of multispecies hpBIOMs composed of *P. aeruginosa* combined with MRSA or *E. faecium*, respectively, identified major differences. While the combination of *P. aeruginosa* and MRSA showed no additional impacts on the model, an accelerated degradation of the model was observed for the combination of *P. aeruginosa* and *E. faecium*. In the histological images of the *P. aeruginosa*–MRSA combination, it was observed that both species remained spatially separated (Figure 3c,d). In the *P. aeruginosa*–*E. faecium* biofilms, bacteria were also located in close proximity (Figure 3a,b). Moreover, there is evidence that *P. aeruginosa* remodels or degrades the hpBIOM starting from the *E. faecium* colonies. In contrast, there is no comparable *P. aeruginosa* degradation in the areas of MRSA colonies.

**Figure 4:**
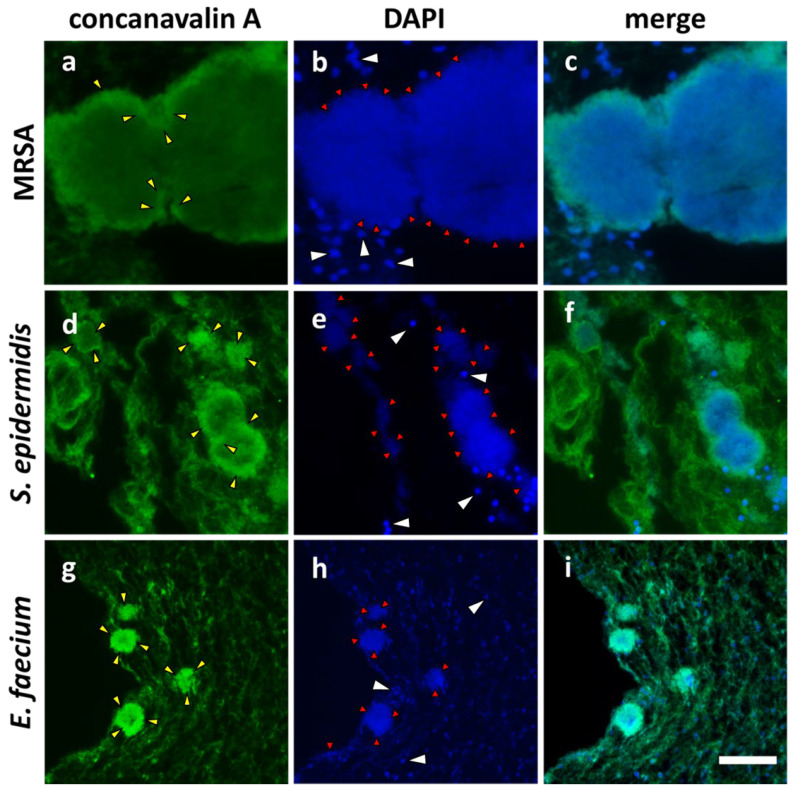
Immunohistochemical staining of the hpBIOM glycocalyx. Carbohydrates were detected using FITC-conjugated Concanavalin A (yellow arrows). Bacterial (red arrows) and cellular nucleic acids (white arrows) were stained with DAPI (blue). MRSA-hpBIOM (48 h) (**a**–**c**), *S. epidermidis* (48 h) hpBIOM (**d**–**f**) and *E. faecium* biofilm (24 h) (**g**–**i**). (Scale bar: 50 µm).

The visualization of the glycocalyx was performed immunohistochemically by means of staining with fluorescence-coupled Concanavalin A (ConA) (Figure 4, green). Nucleic acids from bacteria and immune cells were visualized with DAPI (blue; red arrows). For MRSA, *S. epidermidis*, *E. faecium*, *P. aeruginosa* [43], the formation of a glycocalyx in the hpBIOM was confirmed (yellow arrowheads). Colonies from MRSA and *S. epidermidis* were predominantly surrounded by the glycocalyx. *E. faecium* displayed a more diffuse distribution of the glycocalyx, which was more concentrated within the colonies (Figure 4g–i). Besides these results, the signals of the immunohistochemical analyses revealed clusters, which may lead to the suggestion of the existence of fusion events of MRSA as well as of *S. epidermidis* colonies (Figure 1b,c,h). It was observed that several colonies overlapped, and demarcation between these colonies was hardly possible. Furthermore, the originally condensed surrounding glycocalyx of MRSA and *S. epidermidis* partially disappeared at the presumptive fusion area. Finally, the resulting colony was completely encapsulated by the glycocalyx after fusion (Figure 4c,f).

**Figure 5:**
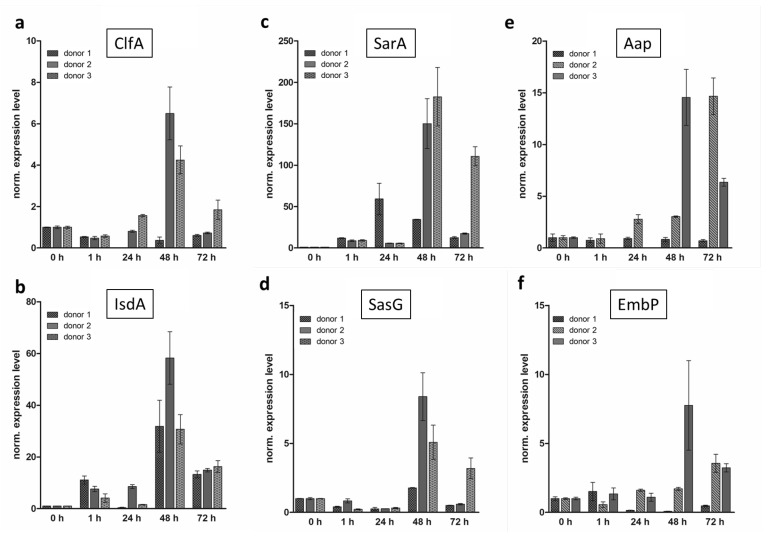
Time-dependent expression of virulence factors in hpBIOMs of three different blood donors. Expression levels of various potential virulence factors isolated in hpBIOMs with MRSA (**a**–**d**) and *S. epidermidis* (**e**,**f**) after 0, 1, 24, 48 and 72 h of maturation (triplets each). (**a**) *ClfA*, (**b**) *IsdA*, (**c**) *SarA*, (**d**) *SasG*, (**e**) *Aap* and (**f**) *EmbP*. (Values expressed as mean ± SD).

In parallel to the histological and immunohistochemical examinations of the hpBIOM, qRT-PCR was performed to evaluate the expression level of various virulence factors. Regarding the time-dependent expression levels, donor-specific effects were detected (Figure 5a–d). In MRSA, all investigated genes were progressively increased to a maximum after 48 h. Subsequently, the level decreased but remained higher compared to the baseline value of the planktonic control (Figure 5a–d). The expression of the fibrinogen-binding proteins *Clfa* (Figure 5a) and *SasG* (Figure 5d) in donors 2 and 3 showed the previously described trend. Donor 1 barely displayed a time-specific expression of *ClfA*. Like donors 2 and 3, a maximum increase in *SasG* expression was detected until 48 h of incubation, but the overall mRNA-level was 3–4-fold lower. For *IsdA*, donors 2 and 3 showed a similar expression profile with a maximum after 48 h, followed by a downregulation after 72 h (Figure 5b). In contrast, donor 1 reached the expression maximum for *IsdA* within 24 h and subsequently decreased again. The expression level of the *SarA* displayed a peak after 48 h. With ongoing maturation, the expression was decreased but slightly remained above the 0 h baseline (Figure 5c). The expression of *IsdA* and *SarA* exceeded the planktonic baseline level within one hour, while the one of *Clfa* and *SasG* passed the baseline initially after 48 h. In *S. epidermidis* hpBIOMs, a strong donor specificity of the expression of *Aap* and *EmbP* was detected (Figure 5e,f). While the expression of *Aap* in donor 1 remained constant and decreased for *EmbP*, in donor 2, the level of both genes enhanced continuously until the maximum was reached after 48 h, followed by a slight downregulation until 72 h of biofilm maturation. The elevation of the expression of *Asap* and *EmbP* in biofilms from donor 3 was delayed with the maximum reached after 72 h (Figure 5e,f).

**Figure 6:**
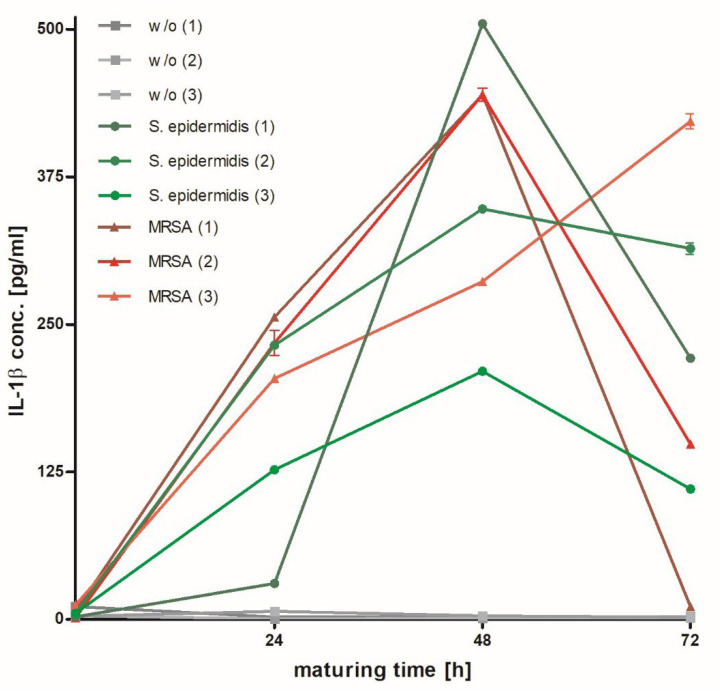
Interleukin 1β (IL-1β) concentration in hpBIOMs after different maturation times. Concentration of IL-1β (pg/mL) in hpBIOMs with MRSA (red), *S. epidermidis* (green) and w/o (gray) after 1, 24, 48 and 72 h of maturation.

By means of ELISA, the concentration of IL-1β, a pro-inflammatory marker secreted by immune cells, was analyzed in hpBIOMs of MRSA, S. *epidermidis* and without a bacterial load after 1, 24, 48 and 72 h (Figure 6). In the control samples, no increase in IL-1β was detected. In contrast, in biofilms produced by MRSA and *S. epidermidis*, the IL-1β concentration was detected during the test period of 72 h. The IL-1β concentration declined after 48 h, except for one donor. Despite some similarities, the IL-1β synthesis was bacteria- and donor-specific.

## 4. Discussion

**Figure 7:**
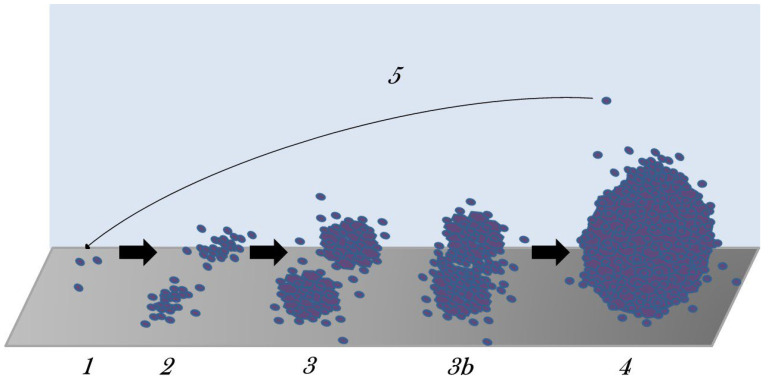
Schematic illustration of biofilm maturation including colony fusion. Well-known biofilm development starting with the attachment of planktonic bacteria to a surface (1), early maturation (2), maturation (3), mature biofilm (4) and finally, the partial disperse of the biofilm to repeat the cycle (5). Additionally, new findings of this study indicate a fusion mechanism of maturing biofilm colonies (3b).

For all tested cocci, MRSA, *S. epidermidis* and *E. faecium*, colony formation was detected within 12 h of maturation (Figure 1). MRSA had markedly larger colonies than the other two species, especially between 12 h and 24 h. Colonization with *E. faecium* resulted in higher degradation of the fibrin matrix of the hpBIOM compared to MRSA or *S. epidermidis*, which prevented the collection and investigation of biofilms after 24 h. Both the growth rate as well as the absolute size of the colonies at the different time points revealed large differences between the tested species. For MRSA and *S. epidermidis*, a decrease in the average diameter was observed between 48 h and 72 h (Figure 1k). This can be explained by the sudden increase in small colonies (0–30 µm in diameter) during this time (Figure 2). This leads to a smaller average size, despite the remaining large colonies. When describing biofilm maturation, spreading, a process where single bacteria detach from the biofilm after advanced maturation and convert back into the planktonic form generating new colonies in another location, represents an important process [49] (Figure 7). The increasing number of small colonies during the maturation of the hpBIOM could be most likely explained by spreading events initiating after 48 h of maturation. This, in turn, suggests that the average maturation time to a mature biofilm requires about two days.

Additionally, donor-specific differences were determined with regard to the bacterial growth rate. Apparently, soluble factors in the plasma or the immune competence have a crucial impact on the proliferation behavior regarding the velocity of growth and the size of the colonies (Figure 2): MRSA colonies of one donor achieved a larger circumference already from 24 h onwards. Other individuals displayed a delayed growth after 72 h or no growth at all. Considering the increase in small colonies between 48 and 72 h, spreading could have occurred about 12–24 h earlier. Presumably, spreading occurs in mature colonies with a diameter of more than 90 µm as well as between 30 and 90 µm. Since an increase in colonies to >90 µm was still recorded for *S. epidermidis* colonies between 48 h and 72 h (Figure 2b), i.e., the absolute colony diameter continued to increase during this period, the maturation time of *S. epidermidis* biofilms is presumably longer than it is for MRSA and thus closer to >60 h. However, in one blood donor, an increase in small colonies could already be seen after 48 h, which at least suggests an earlier spreading time. Considering that no colony formation was observed in approximately ¼ of the *S. epidermidis* hpBIOMs in general, this supports the assumption that the proliferation of *S. epidermidis* bacteria is notably more affected by the donor-specific plasma than MRSA. This is based on the evident higher pathogenicity of MRSA and *P. aeruginosa*, as *S. epidermidis* colonization of a wound is not a negative predictor of wound healing. Irrespective of donor-specific tendencies to influence colony formation in the various species, a synchronous susceptibility to typical antiseptics used in clinical practice is evident [50].

In addition to the histological examination of the hpBIOM, molecular biological investigations were also performed. The expression of the virulence factors *ClfA*, *IsdA*, *SarA* and *SasG* in MRSA as well as the gene expression of *Aap* and *EmbP* in *S. epidermidis* correlated partially with the results of the colony growth observed histologically. It also displayed high donor-specific variations (Figure 5). The expression in hpBIOMs with MRSA predominantly reached their maximums after 48 h (Figure 5a–d) and thus coincided with the maximum average colony diameter (Figure 1k). The expression of *ClfA* (Figure 5a) and *SasG* (Figure 5d) especially increased after 48 h compared to the planktonic expression, suggesting that these proteins are responsible for later processes of maturation. By means of Western blots, it has been documented that *SasG* is presumably not involved in the primary attachment phase but in the accumulation phase of biofilm maturation [51]. *SasG* is believed to mediate intercellular adhesion like Aap in *S. epidermidis* [52]. By comparing the time points of maximum *SasG* and *Aap* expression, these correspond to the maximum colony size of mature colonies of *S. epidermidis* (72 h) and MRSA (48–72 h) and simultaneously reflect the appearance of fusion events (see below). In contrast, *IsdA* (Figure 5b) and *SarA* (Figure 5c) are already up-regulated in the hpBIOM after 1 h of maturation, indicating that these genes are involved in early maturation processes. Nevertheless, the expression also reached a much higher peak after 48 h, which probably implies that these proteins also have an additional function in later maturation processes. Notably, *SarA* is known to regulate a wide range of virulence factors like the genes for a fibronectin-binding protein or enterotoxin C [53,54]. It was possible to reproduce the upregulation of *SarA* compared to the planktonic situation in our model. *IsdA*, which is expressed under iron-limiting conditions is a binding protein with the ability to bind to human ligands [41]. This would explain the high expression as early as 1 h as well as the expected increasing demand with rising cell counts. In turn, the decrease after 72 h is characteristic of quiescence in mature biofilms [55], whereas the new colonies still highly expressed *IsdA*. Maximal growth as well as maximal expression primarily occurs between 24 h and 48 h. The following decrease in the expression after 72 h could be explained, partly, by the general reduction in mature biofilms [10]. Additionally, gene expression after 72 h represents the average of gene expression of the 48 h matured colonies and the new 12 h and younger colonies generated via spreading. Therefore, no predictions can be made about whether gene expression generally decreases after 72 h or is actually sustained in mature colonies but lesser in the < 24 h aged colonies, pulling down the mean value. 

Regarding the expression of *Aap* (Figure 5e) and *EmbP* (Figure 5f) in hpBIOMs of *S. epidermidis*, a significant donor specificity was determined. Both the magnitude and elevation of expression of these two virulence factors seem to depend predominantly on the plasma composition and immune competence of the individuals than on the maturation time (Figure 1k). Again, the comparatively lower virulence of the bacterium has an influence.

As already shown in the histological results, on the gene regulatory level, time- and donor-specific influences were also demonstrated. We also investigated potential immune cell responses in this system by monitoring IL-1β protein levels in hpBIOMs with or without bacteria. IL-1β is a cytokine, a pro-inflammatory mediator, which is secreted by immune cells, for example, to fight bacteria and to trigger an inflammatory response [29]. Molecular analysis of IL-1β concentration in hpBIOM via ELISA revealed donor-specific effects (Figure 6). It was demonstrated that IL-1β increased substantially in the presence of MRSA or *S. epidermidis* compared to the control situation in hpBIOM without a bacterial load. Biofilms of MRSA induced a significantly higher IL-1β synthesis compared to *S. epidermidis* within a 48 h maturation period. However, the highest increase in IL-1β, as well as the smallest increase in IL-1β, was observed in *S. epidermidis*, once again indicating the high donor specificity. Conversely, when IL-1β concentration decreased after 48 h, hpBIOMs with MRSA showed the greatest distribution. In general, IL-1β analysis demonstrated an immune response to bacterial colonization during the entire 72 h observation period. While further wound-healing steps, which require additional stimulus from the host body, like re-epithelialization by the surrounding tissue, cannot be mimicked with the hpBIOM, the initial immune defense provided by the neutrophil immune cells [33,56] introduced from the buffy coat is present (Figure 6). Thus, the immune response, exemplified by IL-1β secretion, as well as the gene regulation results provide supporting evidence that the hpBIOM might be suitable for further investigations of the early interactions between immune cells and bacteria and the corresponding biofilm development.

In the histological evaluation of the hpBIOM, we observed colonies with different morphologies and structures compared to the other colonies. These findings were made in mature *S. epidermidis* colonies after around 48 h as well as in MRSA colonies after 24 h (Figure 1b,c,h). While the majority of cocci colonies have a rather uniform circular appearance (Figure 1d), some colonies displayed an unshaped and composed appearance. These colonies were apparently formed via the fusion of multiple colonies, thus contributing to the rapid colony growth of MRSA and *S. epidermidis* colonies. Besides the histological images, the immunohistochemical analyses of carbohydrate synthesis supported this theory. For all examined cocci (Figure 4), the biofilm-typical glycocalyx was detected [6]. The cocci colonies were surrounded by these carbohydrate-containing structures. In the case of *E. faecium*, they were additionally located within the colonies (Figure 4g). In contrast, the bacterial colonies from MRSA and *S. epidermidis* seemed to be strongly encapsulated by this structure (Figure 4c,f). Besides offering simple mechanical protection, the highly accumulated carbohydrate matrix mediates the inhibited accessibility of antimicrobial substances [15,43,57]. Based on the phenotype of the glycocalyx, indicators of fusion processes could be assigned. Prior to fusion, single colonies were separated by their surrounding glycocalyx. With ongoing progression, the glycocalyx in the area of the fusion edges became degraded. Inside the merged colony, areas with fragments of this carbohydrate barrier were still present (Figure 4a,d). In addition, the lower colony density (Figure 4c,f. DAPI staining) in this area also suggests that the separating glycocalyx was originally located at this region (Figure 4b). The surrounding structure seemed to stay intact during the entire fusion process, which would make sense for its function as a protective layer.

An additional aspect that has been addressed in this work is related to the analysis of hpBIOMs with multiple bacterial species to identify potential synergistic effects. Regarding this, we were able to detect an increase in degradation of the hpBIOM infected with 5 × 10^5^ cfu *P. aeruginosa* and 5 × 10^5^ cfu *E. faecium* compared to 10^6^ cfu of the respective single species. However, these effects were not detected for hpBIOMs with *P. aeruginosa* in combination with MRSA. Histological images showed that *P. aeruginosa* colonies are frequently found at the edge and along cracks and gaps in the fibrin network of the hpBIOM [43]. This correlates with the results of Serra et al. (2015) [58], reporting that *P. aeruginosa* spreads deep in the wound area. In multispecies hpBIOMs, it was observed that *P. aeruginosa* is colocalized with cocci colonies of *E. faecium* at some of these cracks along these fissures (Figure 3a,b). It is possible that *P. aeruginosa* uses these cavities in the fibrin structure formed around the cocci colonies for colonization. The resulting accelerated colonization could lead to faster degradation of the model. In comparison, hpBIOMs with *P. aeruginosa* and MRSA showed that their colonies remain separated (Figure 3c,d). For unknown reasons, *P. aeruginosa*, unlike *E. faecium*, does not colonize the cavities around the MRSA colonies (Figure 3d). Studies on *S. epidermidis* in combination with *S. aureus* demonstrated that *S. epidermidis* secretes small molecules that interfere with biofilm formation by *S. aureus*. It is possible that this is also the case for *P. aeruginosa*, which could explain the physical separation [59].

Based on these findings, there is a strong awareness of the importance of protecting infected wounds from further pathogens, as multispecies biofilms can interact synergistically, aggravating the infection and thus complicating the treatment [60]. The translational approach chosen here with different human donors has the strength to analyze the individuality of the immune response to more (MRSA, *P. aeruginosa*) or less (*S. epidermidis*, *E. faecium*) virulent bacteria. It becomes apparent that with increasing virulence, the immune response and thus the bacterial growth and the expression of the virulence factors become more homogeneous and less donor-specific. The individual biofilm models, on the other hand, have the limitation that the bacterial species examined show greater variation in their interaction. However, with regard to chronic wounds, which also show great diversity in terms of microbial colonization or underlying disease, these donor-specific insights contribute to a more realistic outcome of therapeutic options tested on the model.

## 5. Conclusions

The characterization of the translational biofilm model hpBIOM revealed a high reproducibility of known in vivo results. For example, there was an increase in small colonies after 72 h in comparison to 48 h, which could be explained by spreading events and the protective function of the glycocalyx, which was visualized via immunohistochemical analyses. In addition to the already established knowledge about biofilm growth and spreading, we could detect colony fusion events for MRSA and *S. epidermidis* for the first time. Exploring these fusion events and the underlying mechanism is a relevant subject for further investigation. A comparison group with biofilm-defective mutants could provide further insights and a better understanding of biofilm maturation.

Additionally a broad donor specific range of colony development could be reported. For instance, 25% of all *S. epidermidis* samples formed no visible colonies in the hpBIOMs at all. For *S. epidermidis* as well as for MRSA, a wide range for the distribution of colony diameters could be observed. Similar results were obtained for the expression of several virulence factors for MRSA and *S. epidermidis*. The maximal expression level was determined after 48 h, but a later maximum after 72 h or an early one after 24 h occurred. An analysis of the IL-1β concentration in the hpBIOM revealed an increased concentration in the presence of bacteria. IL-1β is part of the primary immune response, and therefore, these results confirmed the functionality of the immune cells in this model throughout the entire experimental procedure.

Overall, a donor specificity could be observed which, however, has little or no influence on the cytostatic or cytotoxic effects of antimicrobial substances or competing bacterial species. Therefore, individualized medicine needs to be used more frequently for developing new therapies. Especially with regard to the varying results of RT-qPCR, for example, a blockade of fibrinogen-binding molecules could have a positive effect to fight biofilms in wounds in some patients, whereas hardly any difference is to be expected in others.

To sum up, this model is suitable to simulate biofilm maturation during the initial wound-healing process. Moreover, due to the integrated immune components, this model is able to address effects that are lacking in other in vitro models and thus can contribute to novel clinically relevant insights focusing on the individualized medicine approach.

## Figures and Tables

**Table 1:** Primer list. Listed are the used primers (Sigma-Aldrich, St. Louis, MO, USA), the name of the corresponding gene or encoded protein, the sequence of forward (fw) and reverse (rv) primers and the reference.

Primer	Gen/Protein	Sequence	Reference
Reference *S. aureus*	*DNA gyrase subunit β*	fw. TTATGGTGCTGGGCAAATACA rv. CACCATGTAAACCACCAGATA	[45]
Reference *S. epidermidis*	*DNA gyrase subunit β*	fw. CTGACAATGGCCGTGGTATTC rv. GAAGATCCAACACCGTGAAGAC	[25]
*Aap*	*Accumulation associated protein*	fw. TCACTAAACAACCTGTTGACGAA rv. AATTGATTTTTATTATCTGTTGAATGC	[46]
*EmbP*	*Extracellular matrix-binding protein*	fw. AGCGGTACAAATGTCAATATC rv. AGAAGTGCTCTAGCATCATCC	[24]
*Clfα*	*Clumping factor α*	fw. ATTGGCGTGGCTTCAGTGCT rv. CGTTTCTTCCGTAGTTGCATTTG	[47]
*IsdA*	*Iron-regulated surface determinant A*	fw. TGCTTTTTCAAATTCCAAATGCGTAGT rv. GCAGTTGAACCTGGATATAAGAGCTTA	[48]
*SarA*	*Transcriptional regulator SarA*	fw. GGCTTGTTGACTGACTTGTATATGATGA rv. CAAAGTGCCTCAAACTCAACAAGTA	[48]
*SasG*	*Staphylococcus aureus surface protein G*	fw. GTCCATGGAACTTGTATAAATGTATCCAGT rv. GCAGAAGAATATTTAACTAATGGTGGAATCCT	[48]

## Data Availability

The data presented in this study are available upon request from the corresponding author.
